# Basidiomycete-specific chitin synthase genes have clade-specific roles in cell wall formation and hyphal development in Pleurotus ostreatus

**DOI:** 10.1099/mic.0.001651

**Published:** 2026-01-09

**Authors:** Kim Schiphof, Moriyuki Kawauchi, Kenya Tsuji, Akira Yoshimi, Chihiro Tanaka, Shigekazu Yano, Takehito Nakazawa, Yoichi Honda

**Affiliations:** 1 Graduate School of Agriculture, Kyoto University, Kitashirakawaoiwakecho, Sakyo-ku, Kyoto 606-8502, Japan; 2 Graduate School of Global Environmental Studies, Kyoto University, Kitashirakawaoiwakecho, Sakyo-ku, Kyoto 606-8502, Japan; 3 Graduate School of Sciences and Engineering, Yamagata University, Jonan,Yonezawa, Yamagata 992-8510, Japan

**Keywords:** agaricomycete, basidiomycete-specific chitin synthase, cell wall, chitin, *Pleurotus ostreatus*

## Abstract

Chitin, an essential structural component of most fungal cell walls, is produced by transmembrane proteins called chitin synthases (CHSs). Our previous study identified novel basidiomycete-specific *chs*s (*chsb*) clades (BI–BIII) and suggested functional differences between the *chsb* genes of clade BII and BIII. This study, together with our previous work, presents the first comprehensive functional analysis and identification of clade-specific roles of *chsb* genes in vegetative hyphae of the white-rot fungus *Pleurotus ostreatus*. Using homologous recombination, we disrupted *chsb1* (clade BI) and simultaneously disrupted *chsb2* and *chsb3* (clade BII) to investigate their roles in vegetative growth, cell wall biosynthesis and stress response. Deletion of *chsb1* led to reduced colony growth, impaired aerial hyphae formation, thinner cell walls and significant cell wall remodelling. In contrast, single disruptions of *chsb2* or *chsb3* caused mild phenotypes, while double disruption of these genes resulted in severe growth defects, complete loss of aerial hyphae, and abnormal septation. Relative amounts of chitin were increased in the Δ*chsb1* and Δ*chsb2*Δ*chsb3* strains, whereas β-glucan was decreased, which is likely related to cell wall thinning. Together with our previous study, these results reveal clear functional differentiation between *chsb* clades: clade BI influences the relative percentage of cell wall components, as well as radial and aerial hyphal growth; clade BII affects overall growth, cell wall component and septum formation; and clade BIII appears to have a more specific role in aerial hyphae development. These findings advance our understanding of cell wall evolution in filamentous basidiomycetes.

## Introduction

Fungal cell walls play diverse but critical roles in maintaining cell structure and strength, hyphal elongation, fruiting body formation and interactions with the outside environment [1,3]. In filamentous fungi, mycelial cell walls generally consist of chitin, β-1,3-glucan and α-1,3-glucan, as well as various other polysaccharides and proteins [1]. Chitin is a polymer of *N*-acetylglucosamine linked by β-(1,4)-glycoside bonds and can vary in folding, organization, degree of acetylation and orientation [2,3]. Chitin is often embedded deep within the cell wall and crosslinks to β-glucan to form a chitin-glucan complex or anchors cell wall proteins, providing scaffolding for the cell wall, although the amount of chitin can vary between phyla [4,11].

Chitin is critical for survival and is an ancestral fungal cell wall polysaccharide [8,12], and together with its deacetylated form chitosan, they are abundant biological compounds which have applications in plant disease resistance, medicines, biopesticides, agriculture and wastewater treatment [13,14]. Chitin affects mycelial strength and cell wall rigidity, making it a candidate for the production of improved mushroom biomaterials, and *Pleurotus* and *Lentinula* have been gaining attention as sustainable chitin sources [10,15, 16].

Chitin is synthesized by a range of chitin synthases (CHSs). CHSs belong to the glycosyltransferase family 2 (GT2) and polymerize UDP-*N*-acetylglucosamine into UDP and *N*-acetylglucosamine, which subsequently form the cell wall chitin layer. The number of CHSs varies across different species [10,17], and they have a wide range of functions and impacts. Disruption of chitin synthases in some yeasts can be lethal, and in filamentous fungi, disruption impacts range from minor phenotype differences to cell wall component changes, growth impacts, pellet size, sporulation, and virulence [18,24]. Chitin deacetylases and chitinases also play key roles in cell wall chitin regulation and can impact processes such as cytokinesis and stipe cell wall extension [1,3].

In agaricomycetes, numerous *chs* genes have been identified and bioinformatically or enzymatically analysed [17,25,30]. Furthermore, CHS class VI has previously been found to contain no basidiomycete species [3,6], and several studies have proposed the existence of basidiomycete-specific CHSs [7,17, 30], suggesting that these proteins may have lineage-specific roles and offer valuable insights into the evolution of basidiomycete CHS functions and fungal cell wall biology.

Our previous study predicted nine *chs* genes in *Pleurotus ostreatus* and three novel clades of basidiomycete-specific *chs* (*chsb*) genes, with four *chsb*s: *chsb1* (clade BI), *chsb2* and *chsb3* (clade BII) and *chsb4* (clade BIII). Functional analysis of single gene disruptants of *chsb2*, *chsb3* and *chsb4* revealed roles in aerial hyphae formation, cell wall thickness and stress resistance [17]. Distinct phenotypes and relationships between clades BII and BIII suggest that functional differences among CHSBs are associated with their phylogenetic grouping. In this study, we aimed to elucidate clade-specific functions of the *P. ostreatus chsb* genes in vegetative hyphal growth. To do so, single disruptants of *chsb1* (Δ*chsb1*) and double disruptants of *chsb2* and *chsb3* (Δ*chsb2*Δ*chsb3*) were isolated and functionally analysed. Our study highlighted that *chsb1* and *chsb2chsb3* disruption causes severe growth defects and alters relative percentages of cell wall components. Additionally, double disruption of *chsb2chsb3* causes abnormal septation. Elucidating the functions of these genes enhances our understanding of cell wall specialization across fungal phyla and provides new insights into the evolution of basidiomycete cell walls.

## Methods

### Strains, culture conditions and genetic techniques

All *P. ostreatus* strains used in this study are listed in Table S1 (available in the online Supplementary Material). Routine cultures were conducted using 20 ml of yeast extract/malt extract/glucose (YMG) medium [31] solidified with 2% (w/v) agar and incubated at 28 °C in continuous darkness. Strains were precultured for 14 days, and Φ4 mm plugs were used to inoculate.

### Construction of gene disruption strains

Δ*chsb1* disruption strains were generated by *chsb1* targeting through homologous recombination using a hygromycin resistance cassette with the 20b strain as a host [32]. Δ*chsb2*Δ*chsb3* double disruption strains were generated by *chsb2* targeting through homologous recombination using a bialaphos resistance cassette with the Δ*chsb3#2* strain as the host [17]. For *P. ostreatus* transformation, the 20b or the Δ*chsb3#2* strain was cultured in YMG liquid medium with shaking for 4 days, after which protoplasts were prepared from the mycelia using cellulase from *Aspergillus niger* (Sigma-Aldrich, St. Louis, MO, USA), Yatalase -Plus- (Takara Bio, Shiga, Japan) and β-glucanase from *Trichoderma longibrachiatum* (Sigma-Aldrich, St. Louis, MO, USA). DNA disruption cassettes were introduced into the protoplasts using the polyethylene glycol/calcium chloride method, as previously described [32,33].

Gene disruption cassettes were constructed using fusion PCR of three designed DNA fragments: the upstream and downstream regions of the target gene and a drug resistance marker. Genomic DNA of *P. ostreatus* PC9 was used as a template to amplify about 1.5 kb of the 5′ and 3′ flanking regions of *chsb1* and *chsb2* with respective primers (Fig. S1, Table S2). The hygromycin resistance marker (*hph*) used for *chsb1* disruption was amplified from the pPHT1 plasmid [34]. The bialaphos resistance marker (*bar*) used for *chsb2chsb3* double disruption was amplified from the pFNCB plasmid [35] (Fig. S1). Each fragment was amplified using Prime STAR GXL polymerase (Takara Bio, Shiga, Japan) and fused in the second round of PCR. The amplified fragments were purified using a Nucleospin PCR Clean-up and Gel Extraction Kit (Takara Bio, Shiga, Japan) and used as disruption cassettes. Fusion primer schematics and primers are shown in Fig. S1 and Table S2.

### Rapid PCR

PCR was conducted using KOD FX Neo DNA polymerase (TOYOBO, Osaka, Japan) with crude genomic DNA from candidate transformants as a template [36]. The integration of the disruption cassettes was confirmed by PCR amplification of the target gene locus to confirm the presence or absence of the target genes (Figs S2 and S3). Both *hph* and *bar* are more than 2 kb shorter than *chsb1* and *chsb2*; therefore, a second PCR was performed using different primer sets to confirm the length change of the gene locus upon integration of the cassette. The primers and PCR schematics are shown in Fig. S2 and Table S2 and gel electrophoresis results in Table S3.

### Southern blotting analysis

About 3 µg of genomic DNA was extracted from each disruption strain and the 20b strain using the cetyltrimethylammonium bromide method, as described previously [37,38]. Genomic DNA was digested using restriction enzymes, followed by size fractionation in 1% agarose gel and transferred to a positively charged Hybond-N+membrane (Cytiva, Marlborough, MA). Probes were prepared using a PHOTOPROBE (Long Arm) Biotin, Nucleic Acid Labelling Kit (Vector Laboratories, San Jose, CA). Hybridization was conducted using the Amersham AlkPhos Direct Labeling Modules (Cytiva, Marlborough, MA) overnight at 55°C with shaking. Washing buffers were made as described in the manufacturer’s protocol. Primary and initial secondary washing was performed according to the manufacturer’s protocol. After washing twice with secondary wash buffer, 5 µl of Streptavidin Alkaline Phosphatase (Promega, Madison, WI) was added to 10 ml of secondary buffer, mixed and poured onto a plastic sheet, after which the membrane was placed DNA side down and incubated in the dark for 10 min. Five grams of SDS was added to 100 ml of secondary wash buffer and used to wash the membrane twice, after which the secondary wash buffer was used to wash the membrane twice more. CDP-star from the Amersham AlkPhos Direct Labeling Modules (Cytiva, Marlborough, MA) was then added to a plastic sheet, and the membrane was placed DNA side down and incubated in the dark for 2 h, after which chemiluminescence was visualized using an Amersham Imager 680 system (Cytiva, Marlborough, MA). Details of Southern blotting experiments are shown in Fig. S4.

### Growth and morphological observations

Mycelial diameters at 10 days were measured and images were captured using the stereomicroscope SMZ25 (Nikon, Tokyo, Japan) to observe hyphal sparsity.

For aerial hyphae observations, 15 ml of YMG agar was dispensed into test tubes (Φ16 mm), and a mycelium plug was inoculated into the centre of each tube. After 14 days, images were captured to examine the height of the aerial mycelia at the medium–air interface.

For total fungal biomass measurements, 20 ml of YMG liquid medium was added to each plate and a mycelium plug was inoculated into the centre and incubated for 14 days before freeze-drying and weighing.

### Microscopy observations

For transmission electron microscopy (TEM) and scanning electron microscopy (SEM), mycelia at 10 days were used, and observations were made after fixation and dehydration, as described previously [39].

For fluorescent cell wall staining, four mycelium plugs were inoculated into 40 ml of YMG liquid medium and incubated at 28°C for 5 days at 120 r.p.m. Samples were then homogenized using a polytron homogenizer, and 200 µl of the homogenized solution was inoculated into 40 ml of fresh YMG liquid medium and incubated at 28°C, 120 r.p.m. for 2 days. Two millilitres of mycelial solution was then washed with 1 M Tris-HCl, after which 100 µl of 1% Tris-HCl was added to each sample. For CFW staining, 4 µl of 2% calcofluor white stain (Sigma-Aldrich, St. Louis, USA) was then added. For chitin-specific staining, 10 µl of 10 nmol ml^−1^ chitin-binding fluorescent protein (ChBD-GFP) was added [40]. Subsequently, all samples were placed on ice for 2 h with intermittent mixing. Fluorescence microscopy was performed, and images with a Z-stack of 10 µm were taken using the confocal microscopy platform STELLARIS (Leica Microsystems, Wetzlar, Germany). Hyphal thicknesses were measured using the Leica LASX measuring tool.

### Stress and cell wall synthesis inhibitor resistance assay

YMG agar was supplemented with 5 mM H_2_O_2_ and 0.02% SDS to test oxidative and membrane stress resistance, 0.3 M NaCl and 0.3 M KCl to investigate osmotic stress and 100 µg ml^−1^ Micafungin (MF) and 500 µg ml^−1^ Calcofluor White (CFW) to test cell wall stress resistance. Fluorescent Brightener 28 disodium salt solution (Sigma-Aldrich, St. Louis, MO, USA) and Funguard (Astellas, Tokyo, Japan) were used as the CFW and MF, respectively. Non-supplemented YMG was used as a control. A mycelium plug was inoculated into the centre of each plate and incubated for 10 days. The colony diameters of the strains on the supplemented plates (D1) and those on the YMG control (D2) were measured. The relative inhibitory rate was calculated as follows: Relative inhibitory rate (%) = (D2−D1)/D2×100.

### Glucan and chitin measurements

Four mycelium plugs were inoculated into 40 ml of YMG liquid medium and incubated at 28°C for 5 days at 120 r.p.m. Samples were then homogenized using a Polytron homogenizer (Kinematica AG, Malters, Switzerland), after which 1 ml of homogenized sample was inoculated into fresh liquid YMG for Δ*chsb1* and its respective 20b strain, and to ensure adequate mycelial amounts due to severe growth defects, 2 ml was inoculated for Δ*chsb2*Δ*chsb3* and its respective 20b strain and incubated at 120 r.p.m. for an additional 5 days before being homogenized and centrifuged. For every gram of mycelia, 10 ml of fresh YMG was added to ensure consistent mycelial concentrations across all strains. One millilitre of the sample was inoculated onto 20 ml YMG agar plates with added cellophane circles (Φ90 mm). After 14 days of culture, mycelia were harvested and washed with water, ground into a powder using liquid nitrogen and a mortar and pestle and then freeze-dried.

For α- and β-glucan measurements, a β-Glucan Assay Kit (Yeast and Mushroom) (Megazyme, Bray, Ireland) was used to conduct measurements of total glucan and α-glucan in accordance with their respective protocols, using half amounts of reagents and samples. All measurements were conducted in duplicate. Subsequently, relative amounts of α-glucan plus background sucrose and β-glucan were calculated in accordance with the manufacturer’s protocol calculations.

For chitin measurements, 400 µl of 4 N HCl was added to 0.01 g of ground mycelia and heated at 96°C for 16 h before adding 400 µl of water to cool the reaction. Ground activated charcoal was added to each sample and vortexed three times over 1 h, after which samples were centrifuged and 300 µl of supernatant was diluted tenfold using 2.63 ml water and 70 µl of 8 N NaOH to neutralize the sample. The relative percentage of chitin after extraction was determined using a d-Glucosamine Assay Kit (Megazyme, Bray, Ireland) in accordance with the manufacturer’s protocol.

### Quantitative reverse transcription PCR

Mycelia were cultured in the same manner as for glucan and chitin measurements. After 14 days of culture, mycelia were harvested and ground into a powder using liquid nitrogen and a mortar and pestle. A FastGene RNA Premium Kit (NIPPON Genetics, Tokyo, Japan) was used to extract RNA. The obtained RNA samples were reverse transcribed using PrimeScript RT Master Mix (Takara Bio, Shiga, Japan). PowerTrack SYBR Green Master Mix and QuantStudio 5 (Thermo Fisher Scientific) were used for quantitative reverse transcription PCR. Quantification was performed as previously described [41]. Within each independent biological replicate, quantitative reverse transcription-PCR was conducted in technical duplicates and mean Ct values were used for calculations. The primer pairs used for the amplification of the cDNA fragments and their amplification efficiencies are listed in Table S4.

### Bioinformatic and statistical analysis

Bioinformatic analysis to identify *chs* genes and clades was conducted in our previous study [17] using blastp searches. Three independent biological rounds (*n*=3) were conducted for all experiments. Significant differences between 20b and the *chsb* disruption strains were investigated using two-tailed Student’s *t*-tests assuming equal variance.

## Results

### Single disruption of *chsb1* and double disruption of *chsb2chsb3* causes severe defects in growth and aerial hyphae formation

Gene targeting via homologous recombination using the 20b and Δ*chsb3* strain as a host was utilized to knock out the entire gene locus and isolate single disruptants of *chsb1* (clade BI) and double disruptants of *chsb2chsb3* (clade BII), respectively (Fig. S3). All candidates were confirmed using genomic PCR and two independent strains (Δ*chsb1*#5 and Δ*chsb1*#6, and Δ*chsb2*Δ*chsb3*#3 and Δ*chsb2*Δ*chsb3*#5) were selected and used alongside the 20b strain for subsequent experiments. Southern blotting confirmed single integration of each cassette (Fig. S4).

**Fig. 1.**
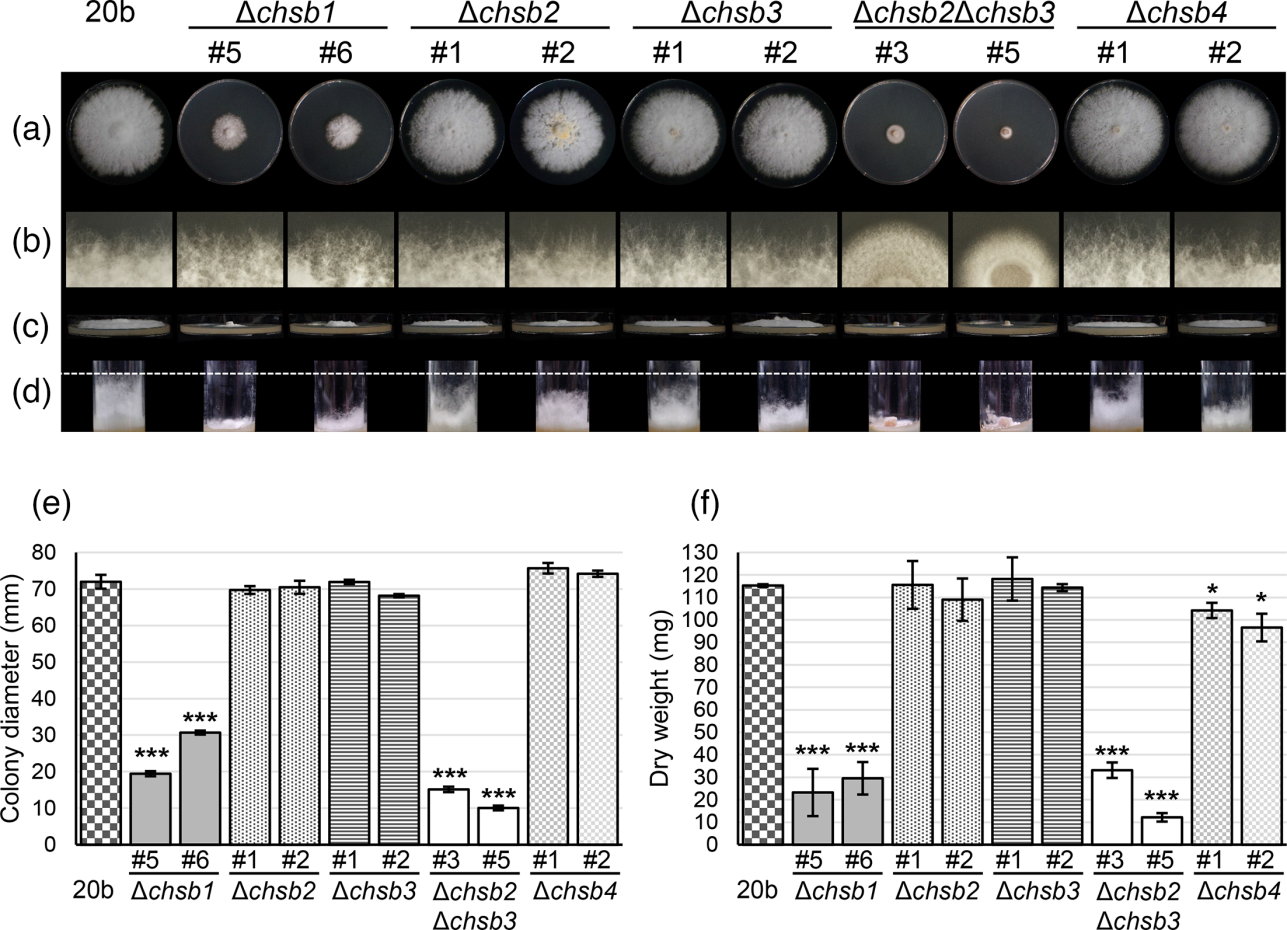
Morphology, growth rate and aerial hyphae assay of the parent strain 20b and *chsb* disruption strains. Effect of disruption on growth morphology of mycelium on YMG agar (**a**) growth morphology, (**b**) mycelium under stereomicroscopy and (**c**) side view of hyphae on plates. (**d**) Aerial hyphae formation at 14 days. The white dotted line indicates the aerial hyphae height of the 20b strain. (**e**) Colony diameter on YMG agar at 10 days, (**f**) dry weight biomass on YMG liquid at 14 days. All bars indicate the standard deviations of three biological replicates (*n*=3). Statistical significance was determined by a two-tailed equal variance *t*-test (∗*P*<0.05, ∗∗*P*<0.01, ∗∗∗*P*<0.001).

Observation and measurement of growth phenotypes on YMG agar plates highlighted significant and severe colony diameter impacts in Δ*chsb1* and almost no radial growth in Δ*chsb2*Δ*chsb3,* which were not observed in any of the *chsb* single disruption strains (Fig. 1a and b). The Δ*chsb1* strains exhibited little aerial hyphae formation compared to the 20b strain, whereas the Δ*chsb2*Δ*chsb3* strains formed almost no aerial hyphae and single disruptants Δ*chsb2*, Δ*chsb3* and Δ*chsb4* exhibited some aerial hyphae formation defects but to a lesser extent (Fig. 1c and d). Furthermore, the Δ*chsb1* and Δ*chsb2*Δ*chsb3* strains exhibited a significant and severe decrease in both colony diameter and dry weight, and despite no change in colony diameter, dry weight biomass of Δ*chsb4* was also slightly decreased, suggesting a hyphal density decrease (Fig. 1e and f). Therefore, all basidiomycete-specific CHSs impact aerial hyphae formation, and clades BI and BII are additionally important for vegetative growth.

### Disruption of *chsb1* and *chsb2chsb3* causes decreased sensitivity and in some cases complete inhibition in response to cell stressors

**Fig. 2.**
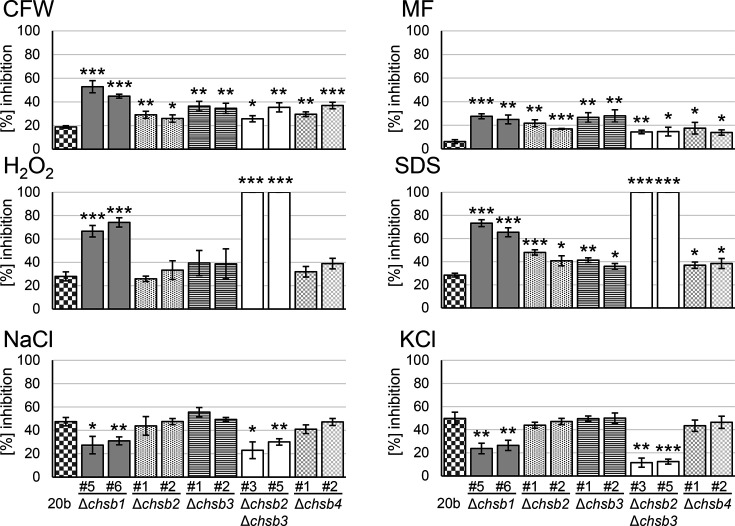
Relative growth inhibition rates of the parent strain 20b and *chsb* disruption strains grown on YMG agar medium supplemented with 5 mM H_2_O_2_, 0.02% SDS, 100 µg ml^−1^ MF, 500 µg ml^−1^ CFW, 0.3M NaCl and 0.3M KCl for 10 days. Bars indicate standard deviations of three biological replicates (*n*=3). Statistical significance was determined using a two-tailed equal variance *t*-test (∗*P*<0.05, ∗∗*P*<0.01, ∗∗∗*P*<0.001).

Sensitivity to CFW, a chitin synthesis inhibitor; MF, a β-glucan synthesis inhibitor; H_2_O_2,_ an oxidative stressor; SDS, a detergent that damages the plasma membrane; and NaCl and KCl, osmotic stressors, were tested (Figs 2 and S5). When compared to the 20b strain, all *chsb* disruption strains were significantly more sensitive to CFW, MF and SDS. The Δ*chsb1* strains were additionally more sensitive to H_2_O_2_, and the Δ*chsb2*Δ*chsb3* strains were completely unable to grow in the presence of H_2_O_2_ and SDS. These results imply cell wall integrity defects, especially in the Δ*chsb2*Δ*chsb3* strains. Furthermore, the Δ*chsb1* and Δ*chsb2*Δ*chsb3* strains were less sensitive to NaCl and KCl, with no changes in sensitivity observed in the other *chsb* disruption strains.

### Microscopy indicates that disruption of *chsb1* and *chsb2chsb3* impacts cell wall thickness and *chsb2chsb3* double disruption additionally impacts septum distribution

**Fig. 3.**
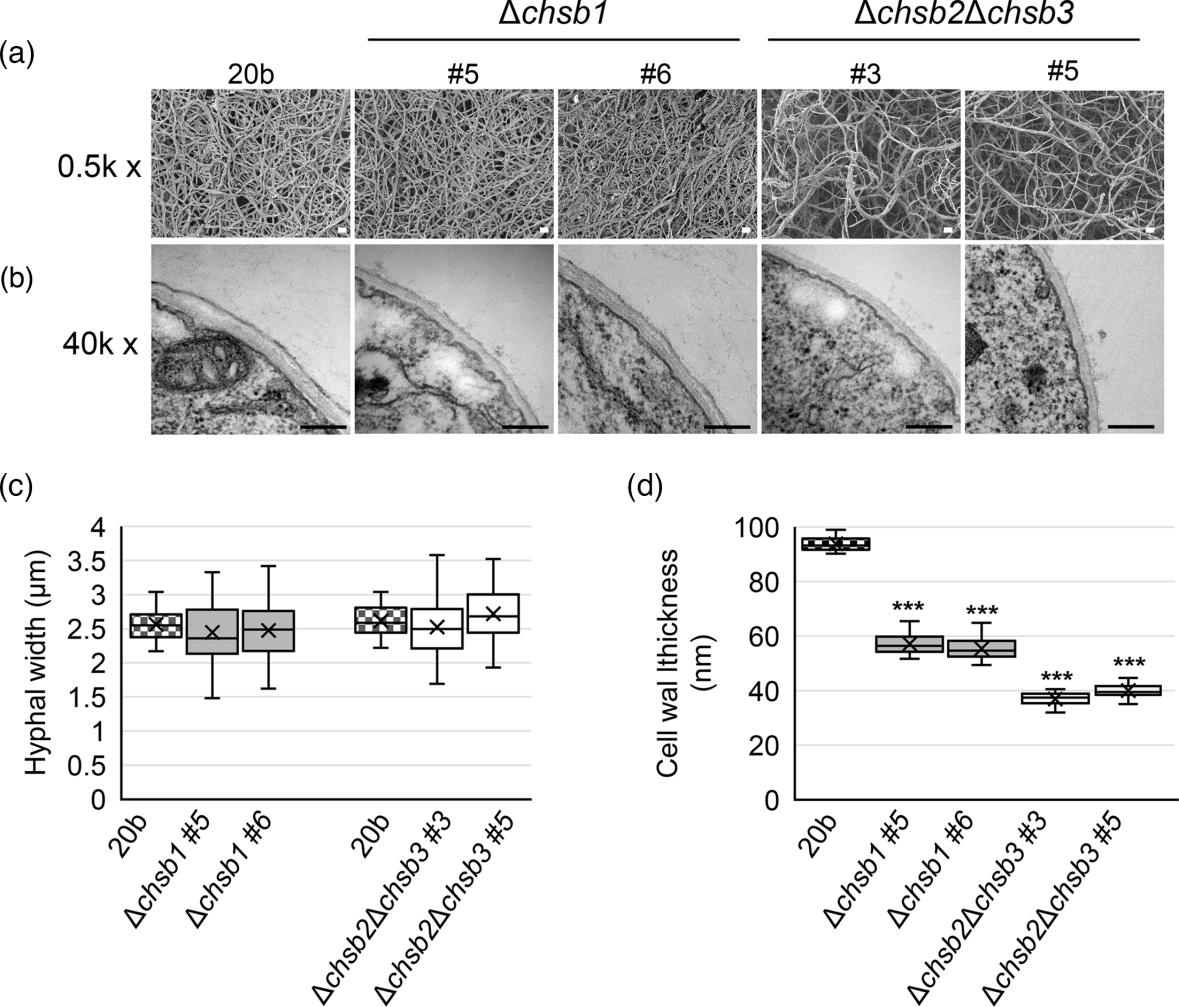
Cell wall and hyphal morphologies of the parent strain 20b and *chsb1* and *chsb2chsb3* disruption strains (left: *chsb1*; right, *chsb2chsb3*). (**a**) SEM, scale bar=10 µm. (**b**) TEM, scale bar=200 nm. (**c**) Hyphal thickness obtained from 20 hyphae across three biological replicates (*n=*60). (**d**) Cell wall thickness obtained from TEM observations in (**b**) (*n*=50*)*. Statistical significance was determined using a two-tailed equal variance *t*-test (∗∗∗*P*<0.001).

SEM revealed that the Δ*chsb1* and Δ*chsb2*Δ*chsb3* strains had inconsistent hyphal thickness and that the hyphae in the Δ*chsb2*Δ*chsb3* strains were seemingly less dense than those of the 20b strain (Fig. 3a). Hyphal thickness measurements from confocal microscopy confirmed that Δ*chsb1* and Δ*chsb2*Δ*chsb3* have inconsistent hyphal thicknesses, with hyphae ranging from 1.7 to 3.5 µm, whereas hyphal thickness of the 20b strain is between 2.2 and 3.1 µm (Fig. 3c). TEM also highlighted significantly thinner cell walls with a decrease of 37% in the Δ*chsb1* strains and 58% in Δ*chsb2*Δ*chsb3* double disruption strains (Fig. 3b, d).

**Fig. 4.**
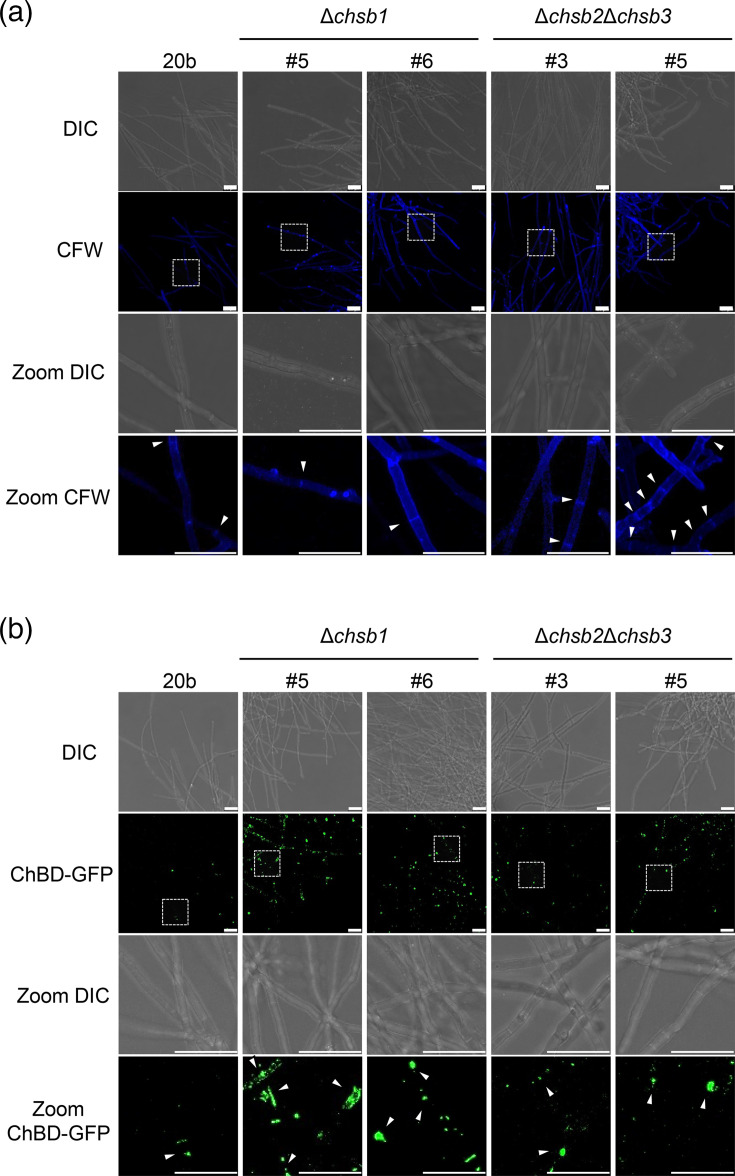
Chitin localization in mycelia of *chsb1* and *chsb2chsb3* disruption strains under a confocal microscope. (**a**) CFW staining, arrows indicate septa. (**b**) Chitin-specific fluorescent probe staining (ChBD-GFP), arrows indicate areas of increased chitin exposure (scale bar=20 µm). 20b, parent strain 20b; DIC, differential interference contrast; Merge, the merged DIC and staining images; Zoom, enlargement of white dotted boxes from merged images.

CFW and chitin-specific probing highlighted the presence of chitin in tips and septa of all strains (Fig. 4a). The Δ*chsb2*Δ*chsb3* strains had abnormal hyphal septa distribution compared to the 20b strain with septa appearing in clusters of at least two septa, non-uniformly distributed along the hyphae (Fig. 4a). Both disruption strains, but especially the Δ*chsb1* strains, had many additional spots of chitin across the hyphal surface compared to the 20b strain (Fig. 4b). These results indicate that *chsb1* is important for cell wall thickness and *chsb2chsb3* for cell wall thickness and septum formation or cell elongation.

### Disruption of *chsb1* and *chsb2chsb3* impacts the relative amounts of cell wall components and causes differential expression of various cell wall synthases

**Fig. 5.**
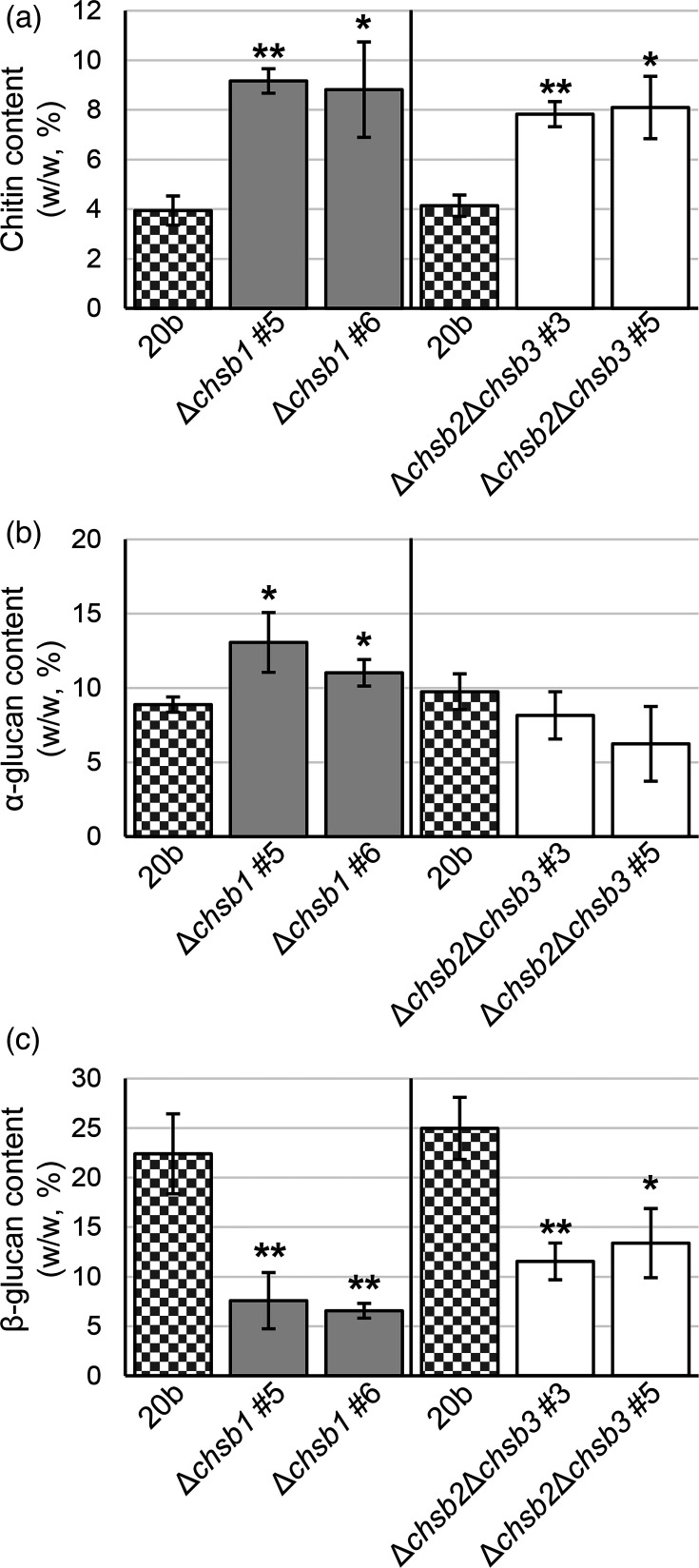
Cell wall polysaccharide analysis of the parent strain 20b and disruption strains (left, Δ*chsb1;* right, Δ*chsb2*Δ*chsb3*). Percentage of mycelial dry weight made up of (**a**) chitin, (**b**) α-glucan and (**c**) β-glucan from mycelia on YMG cellophane at 14 days old. The bars indicate the standard deviations of three biological replicates (*n*=3). Statistical significance was determined using a two-tailed equal variance *t*-test (∗*P*<0.05, ∗∗*P*<0.01, ∗∗∗*P*<0.001).

Chitin content (g/100 g dried mycelia) doubled in both the Δ*chsb1* and Δ*chsb2*Δ*chsb3* strains (Fig. 5a). In the Δ*chsb1* strains, α-glucan (g/100 g dried mycelia) was slightly increased (Fig. 5b). Relative β-glucan content (g/100 g dried mycelia) decreased in both the Δ*chsb1* and Δ*chsb2*Δ*chsb3* strains (Fig. 5c).

**Fig. 6.**
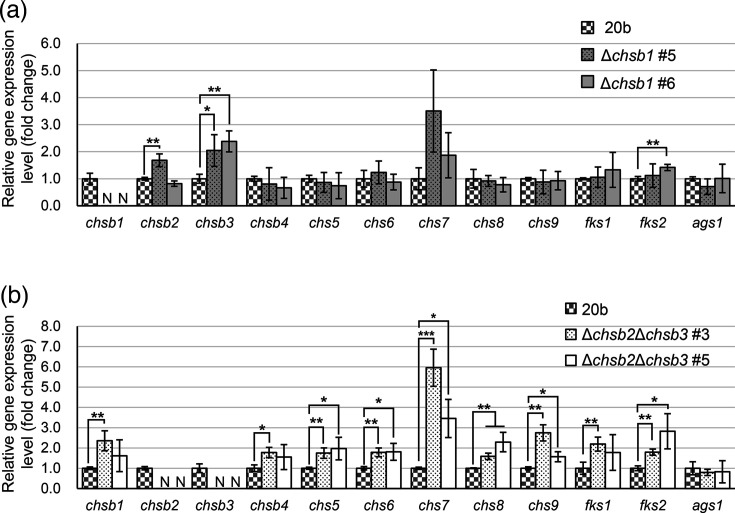
Relative gene expression levels of various cell wall synthases in (**a**) Δ*chsb1* and (**b**) Δ*chsb2*Δ*chsb3*. The bars indicate the standard deviations of three biological replicates (*n*=3). Statistical significance was determined using a two-tailed equal variance *t*-test (∗*P*<0.05, ∗∗*P*<0.01, ∗∗∗*P*<0.001; N, gene expression not detected).

Relative gene expression levels of all nine *chs* genes, two β-glucan synthases (*fks*s) and a single α-glucan synthase (*ags*) were examined (Fig. 6). In both the Δ*chsb1* and Δ*chsb2*Δ*chsb3* strains, *chs7* had the highest relative expression level. In the Δ*chsb2*Δ*chsb3* strains, the *chs7* expression level was more than three times that of the 20b strain. The Δ*chsb2*Δ*chsb3* strains also exhibited increased expression levels for *chsb1*, *chs5*, *chs6*, *chs8*, *chs9* and *fks2,* although these were only slightly upregulated between 1.5- and 2.5-fold higher. The Δ*chsb1* strains also had a twofold increase in *chsb3* expression, compared to the 20b strain. Therefore, clade BI and BII CHSs are critical for cell wall component composition and impact relationships with other cell wall synthases, suggesting attempted compensatory mechanisms.

## Discussion

In our previous study, basidiomycete-specific chitin synthase clades were predicted [17]. However, clade-specific functions of these *chsb*s remain to be elucidated, but may have important and specific roles that could help us understand agaricomycete and CHS evolution. In this study, the clade-specific roles of the *P. ostreatus chsb*s in vegetative hyphae were investigated through the disruption of *chsb1*, the single gene in clade BI (Δ*chsb1*) and double disruption of both genes in clade BII (Δ*chsb2*Δ*chsb3*).

Single disruption of *chsb2*, *chsb3* or *chsb4* does not affect radial growth, but does result in a small decrease in aerial hyphae height, while *chsb1* disruption affects radial growth and causes more severe aerial hyphae defects (Fig. 1), highlighting that single disruption of any *P. ostreatus chsb* gene leads to impaired aerial hyphae development. Notably, Δ*chsb1* is the only single disruption strain that impacts both aerial hyphae and mycelial expansion, pointing to a distinct functional role for clade BI *chsb*s (Fig. 1). In contrast, disruption of *chsb4* (clade BIII), despite being a single-copy gene like *chsb1*, only impacted aerial hyphae formation without impacting radial growth (Fig. 1), suggesting clade BIII *chsb*s serve a more specialized function in aerial hyphae production. Double disruption of *chsb2chsb3* caused severe growth defects and disabled the ability to form aerial hyphae, while single disruptions of *chsb2* and *chsb3* had some small aerial hyphae defects but no changes to colony diameter, highlighting the critical and possibly redundant roles of clade BII *chsb*s in supporting aerial hyphae development.

Fungal cell walls are important for structural integrity, environmental interactions and protection from stress, and chitin plays a key role in maintaining rigidity and resistance to external stressors [1,42,44]. All *chsb* disruption strains were significantly more sensitive to MF, CFW and SDS, with the Δ*chsb1* strains additionally more sensitive to H_2_O_2_, and the Δ*chsb2*Δ*chsb3* strains lethally sensitive to SDS and H_2_O_2_, suggesting severe cell wall defects. Interestingly, despite decreased tolerance to oxidative and cell wall stressors, Δ*chsb1* and Δ*chsb2*Δ*chsb3* were less sensitive to osmotic and ionic stressors. This could be due to increased chitin content or cell wall remodelling, preventing salts from entering the cell wall and allowing for osmotic protection, despite causing other growth and subsequently oxidative and cell wall defects. TEM and SEM further revealed that the Δ*chsb1* and Δ*chsb2*Δ*chsb3* strains have thinner cell walls and display inconsistent hyphal diameters (Fig. 3), which likely contribute to the observed growth and morphological defects.

The morphological defects in the Δ*chsb1* and Δ*chsb2*Δ*chsb3* strains may arise from underlying alterations in cell wall organization, composition and gene regulation. Although chitin synthases in ascomycetes are associated with chitin production at tips and septa, and disruption of these genes can lead to altered localization [5,45,47], single disruption of *P. ostreatus chsb* genes did not affect CFW staining patterns at these sites, although single disruptants of Δ*chsb2* and Δ*chsb3* showed reduced CFW binding capacity [17]. Similarly, chitin was also still present in septa and tips of Δ*chsb2*Δ*chsb3* strains, but these strains displayed abnormal septation with irregularly spaced clusters of septa, possibly due to cell elongation defects (Fig. 4a). Septa can restrict or enable the exchange of cytoplasmic constituents and are crucial for hyphal integrity, nutrient distribution and environmental adaptability [48,51]. Therefore, the disrupted spatial organization of septa in Δ*chsb2*Δ*chsb3* may be related to their impaired growth.

**Fig. 7.**
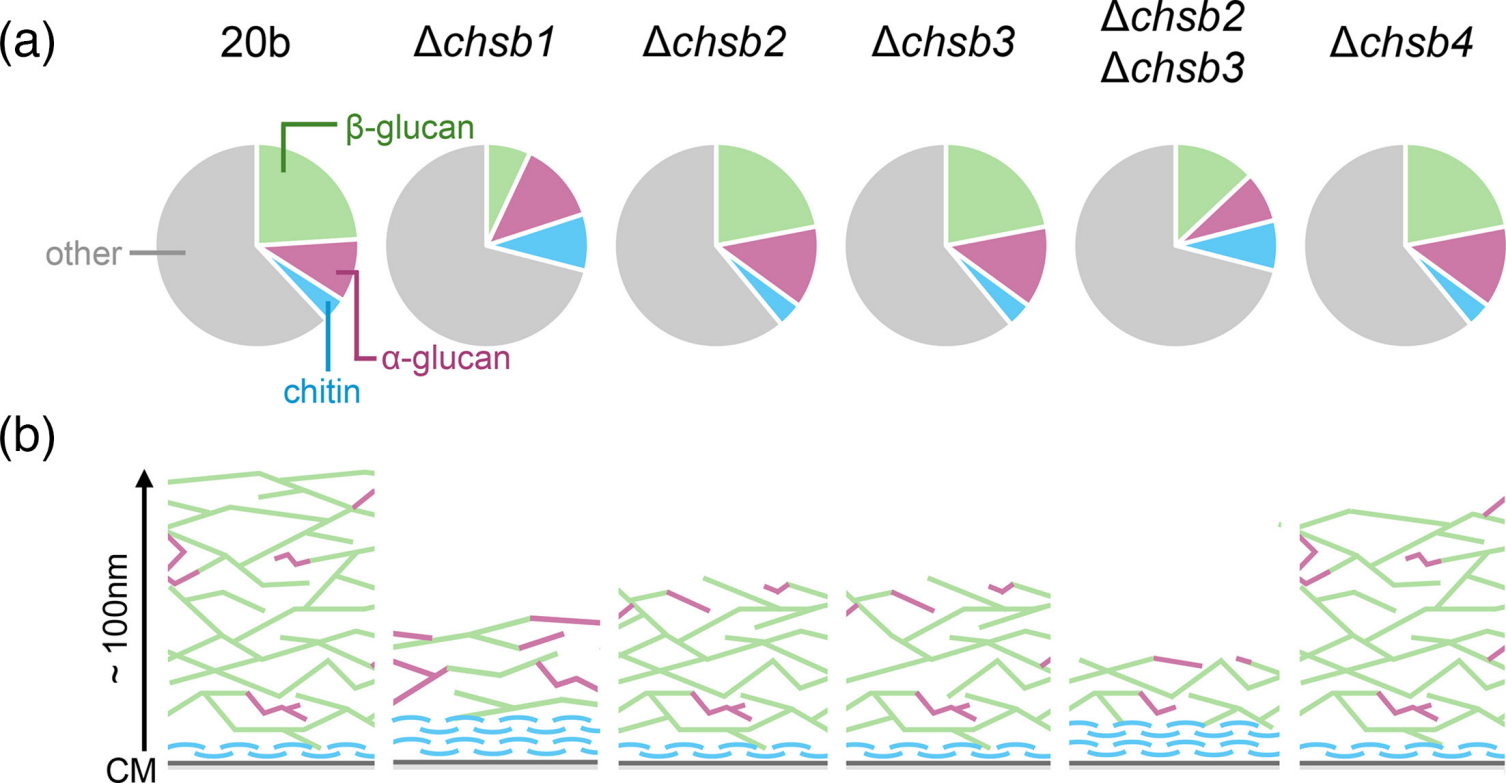
Summary of experimental results of this study and our previous study [17] and schematic diagram of hypothesized inner cell wall remodelling in response to basidiomycete-specific chitin synthase disruption in *P. ostreatus*. (**a**) Relative percentages of cell wall components. (**b**) Summary of cell wall thickness and component data. Green, β-glucan; pink, α-glucan; blue, chitin; 20b, parent strain 20b; CM, cell membrane. Arrow scale bar=100 nm.

Furthermore, chitin-specific probing revealed increased amounts of chitin-stained spots on the hyphal surface in both the Δ*chsb1* and Δ*chsb2*Δ*chsb3* strains, potentially due to a thinner β-glucan layer, exposing more of the inner chitin layer (Fig. 4b) [10]. Compositionally, these disruption strains displayed altered relative percentages of cell wall components (Fig. 5) with the 20b strain consisting of ~25% β-glucan and ~4% chitin, Δ*chsb1* 7% and 9% and Δ*chsb2*Δ*chsb3* 13% and 8% β-glucan and chitin, respectively. These altered percentages correlate with the increased spots of chitin observed in fluorescence microscopy. A higher chitin proportion can stiffen the cell wall and restrict hyphal elongation [5,13, 52,54], which may contribute to the growth defects seen in the Δ*chsb1* and Δ*chsb2*Δ*chsb3* strains. The Δ*chsb1* strains also showed a slight increase in α-glucan, possibly as a compensatory response. However, given the significant reduction in cell wall thickness and that chitin content was measured as a relative percentage of total cell dry weight, the apparent increase in chitin may be misleading. The absolute amount of chitin may be reduced or equivalent to that of the 20b strain; however, as the cell wall is much thinner in the disruption strains and β-glucan amounts are significantly decreased, chitin makes up a larger percentage of the remaining cell wall. This decrease in β-glucan may be due to altered cell wall composition and chitin production, impairing binding and formation of chitin-glucan complexes. This could explain why, despite upregulation of *fks* genes in the Δ*chsb2*Δ*chsb3* strains, the overall percentage of β-glucan was significantly decreased. Furthermore, compensatory upregulation of cell wall chitin in response to a lack of β-1-3-glucan has been observed in a range of ascomycete fungi [55]. A similar relationship may be occurring in *P. ostreatus* with an increase in chitin causing a decrease in β-glucan (Fig. 7).

Quantitative reverse transcription PCR analysis further revealed that disruption of *chsb1* causes upregulation of *chs7* and *chsb3* (Fig. 6a). Conversely, in *chsb2, chsb3* and *chsb4* single disruption strains, *chsb1* is upregulated [17]. Double disruption of *chsb2* and *chsb3* causes upregulation of *chs7* as well as *chs5*, *chs6*, *chs8*, *chs9* and *fks2* (Fig. 6b). Despite these compensatory responses, the double disruption led to severe growth defects, likely because clade BII *chs*s (*chsb2* and *chsb3*) are functionally redundant within their clade but cannot be adequately compensated for by *chs*s from other clades. Interestingly, *chsb1* (clade BI) and *chsb4* (clade BIII) are upregulated in Δ*chsb2* and Δ*chsb3* (clade BII) single disruption strains but not in Δ*chsb2*Δ*chsb3* double disruption strains. These results imply that clade BII has inter-clade relationships that are dependent on the presence of at least one clade BII *chsb*. The distinct expression pattern of Δ*chsb4* [17], with upregulation of only *chs7*, further supports functional and regulatory divergence among *chsb* clades. Transcription factors in the *P. ostreatus* cell wall integrity signalling pathway also regulate the *chsb* clades independently. Disruption of transcription factors *mbp1* and *swi6* causes upregulation of *chsb3* but no change to *chsb2*, suggesting possible redundancy within clade BII, as well as down-regulation of *chsb1* (clade BI) and *chsb4* (clade BIII) [56,57]. Further investigation on clade-specific compensation mechanisms should be conducted to properly elucidate regulatory divergence amongst *chsb* clades.

In ascomycetes, CHSs play varied roles. In *Saccharomyces cerevisiae*, disrupting all three CHSs is generally non-viable [18], and in *Candida albicans*, inactivation of CaCHS1 is only temporarily viable [19]. In filamentous species like *Aspergillus fumigatus*, some CHSs affect morphology without altering chitin levels [20], while in *Aspergillus niger*, different CHSs influence growth, chitin content, drug resistance or pellet size [21]. In basidiomycetes, CHSs also have diverse roles. In the plant pathogen *Ustilago maydis*, *chs5* and *chs7* impact cell shape, while *chs6*, *chs7* and *chs1* are involved in virulence [22]. In the yeast-like basidiomycete *Cryptococcus neoformans*, *chs3* is involved in growth, chitosan production, and host immune responses [23,24]. The functions of *P. ostreatus chsb* genes seem to be similarly diverse, but with functional differentiation between clades. However, all disruption strains impact aerial hyphae formation, even if colony diameters are unaffected, suggesting some overlapping functions between these genes.

Functional analysis of all basidiomycete-specific chitin synthase clades in *P. ostreatus* reveals clear functional differentiation between clades. Disruption of *chsb1* (clade BI) caused marked growth defects, reduced cell wall thickness and altered relative percentages of chitin and glucan. In contrast, single disruption of *chsb2* and *chsb3* (clade BII) [17] had minimal effects, while double disruption of these genes led to severe growth defects, altered septum formation and cell wall remodelling, suggesting redundancy within clade BII. Disruption of *chsb4* (clade BIII) (Fig. 1) [17] only impacted aerial hyphae formation, without impacting radial growth, suggesting a specific function of clade BIII *chs* genes in aerial hyphae formation. *chs* disruption often triggers compensatory overexpression, leading to increased chitin synthesis [44,58,60]. The altered chitin distribution of Δ*chsb1* and Δ*chsb2*Δ*chsb3* is likely related to cell wall thickness decreases rather than transcriptional compensation. These findings highlight that chitin accumulation alone does not guarantee structural integrity and suggest that different *chsb* clades contribute to cell wall assembly in clade-specific ways. Orthogroups of agaricomycete chitin synthase genes are upregulated in primordia [30], and we previously identified *chsb3* as the homologue to one of these highly expressed genes [17], suggesting important roles of *chsb*s not just in vegetative hyphae, but also in fruiting body formation. Further investigation into the roles of *chsb*s on sexual development and the roles of the remaining *P. ostreatus* chitin synthases is ongoing. It is also of interest in future studies to further investigate other cell wall synthesis-related genes, such as chitin deacetylases and chitinases, through transcriptome analyses with these strains to determine if chitin synthase disruption also affects chitin degradation or deacetylation.

This study emphasizes clade-specific *chsb* functions in the vegetative hyphae of *P. ostreatus* and, together with our previous study [17], demonstrates the value of integrating phylogenetic identification with functional analysis to understand cell wall biosynthesis and its evolution in filamentous fungi.

## Supplementary material

10.1099/mic.0.001651 Uncited Supplementary Material 1.

## Abbreviations

CFW: Calcofluor White
CHSs: chitin synthases
MF: Micafungin
SEM: scanning electron microscopy
TEM: transmission electron microscopy
YMG: yeast extract/malt extract/glucose
