# Supplementary material for: Basidiomycete-specific chitin synthase genes have clade-specific roles in cell wall formation and hyphal development in Pleurotus ostreatus
Source: Microbiology (Reading). 2026 Jan 9;172(1):001651. doi: 10.1099/mic.0.001651 (PMC13293301; doi:10.1099/mic.0.001651)
Supplement: Uncited Supplementary Material 1. [file mic-172-01651-s001.pdf]

Supplementary data

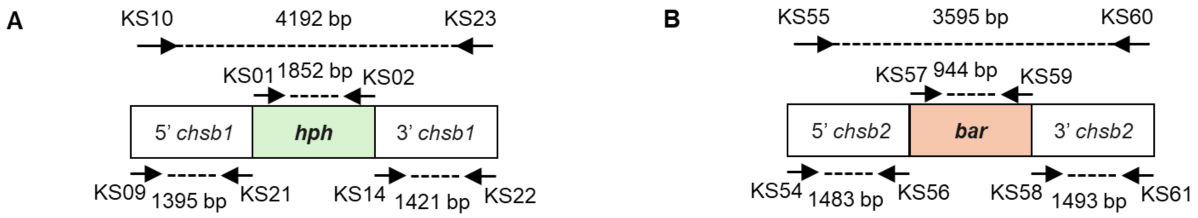

Fig. S1. Gene disruption cassette schematics of (A) Δ*chsb1* single gene disruption. (B) Δ*chsb2*Δ*chsb3* double gene disruption. Primers used for cassette construction are indicated by arrows. Primer names and length of polymerase chain reactions (PCR) products are also indicated. Primer sequences are listed in Table S2.

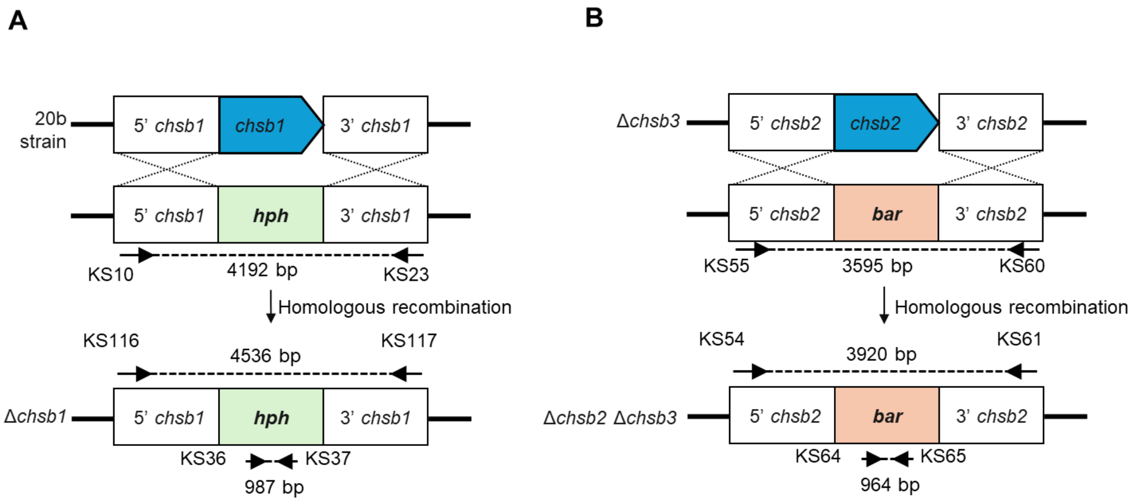

Fig. S2. Schematic of gene disruption of (A) *chsb1,* (B) Δ*chsb2*Δ*chsb3*. Primers used for confirmation are indicated by arrows. Primer names and lengths of expected polymerase chain reactions (PCR) products are also indicated. Primer sequences are listed in Table S2.

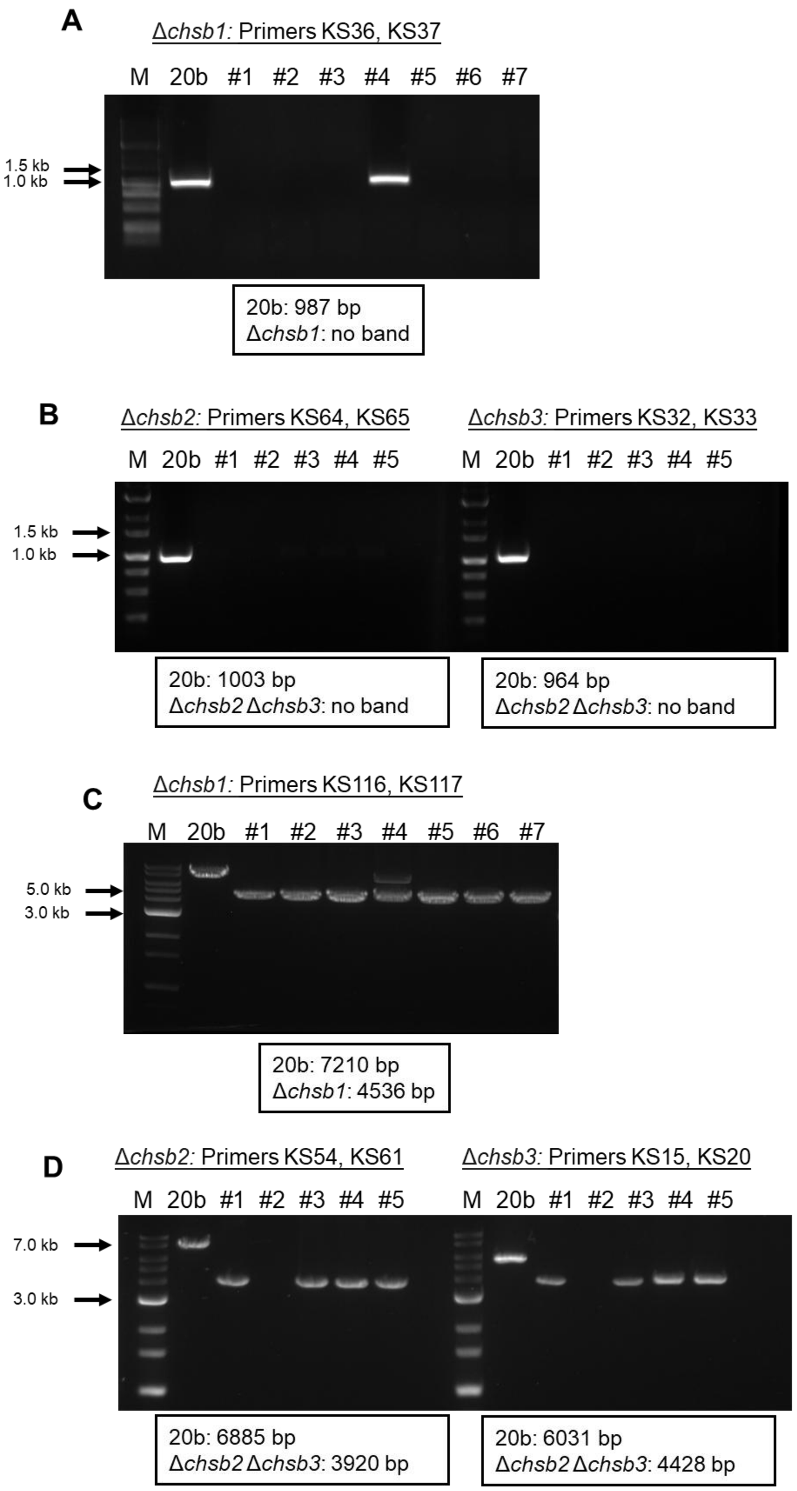

Fig. S3. Polymerase chain reaction (PCR) confirmation of gene disruption. (A) *chsb1* gene presence. (B) Δ*chsb2*Δ*chsb3* gene presence. Left: *chsb2*. Right: *chsb3* using same template DNA. (C) *chsb1* length change*,* (D) Δ*chsb2*Δ*chsb3* length change. Left: *chsb2*. Right: *chsb3* using same template DNA. Primer names for each PCR and ladder length are also indicated. Lane M: 1 kb ladder (Nippon gene, Tokyo, Japan). Lane 20b: parent strain 20b.

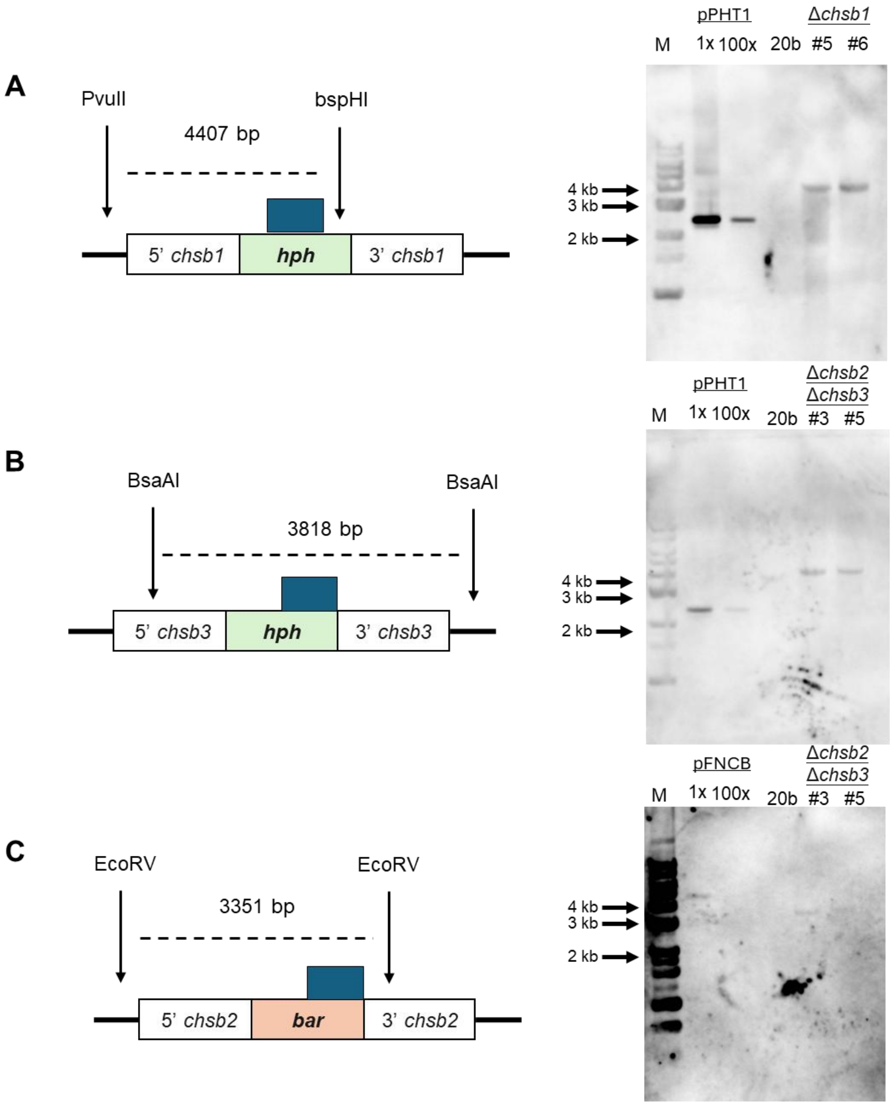

Fig. S4. Schematic diagram and results for Southern blotting analysis (A) *chsb1* disruption strains, (B) *chsb2chsb3* double disruption strains testing for *hph*, and (C) *chsb2chsb3* double disruption strains testing for *bar*. *hph*: hygromycin resistance fragment. *bar*: bialaphos resistance fragment. M: marker. pPHT1: plasmid harbouring *hph* in a 2267 bp fragment. pFNCB: plasmid harbouring *bar* in a 4350 bp fragment. 20b: parent strain 20b. Blue boxes indicate the probe location. Restriction enzyme names and length of expected digestion fragment are indicated. For probes, a partial *hph* fragment (1012 bp) was amplified from the pPHT1 plasmid (1) using primers TK203 and TK204 and a partial *bar* fragment (551 bp) was amplified from the pFNCB plasmid (2) using primers KS126 and KS127. 1×: 3 µg, 100×: 30 ng of the plasmids (pPHT1 or pFNCB).

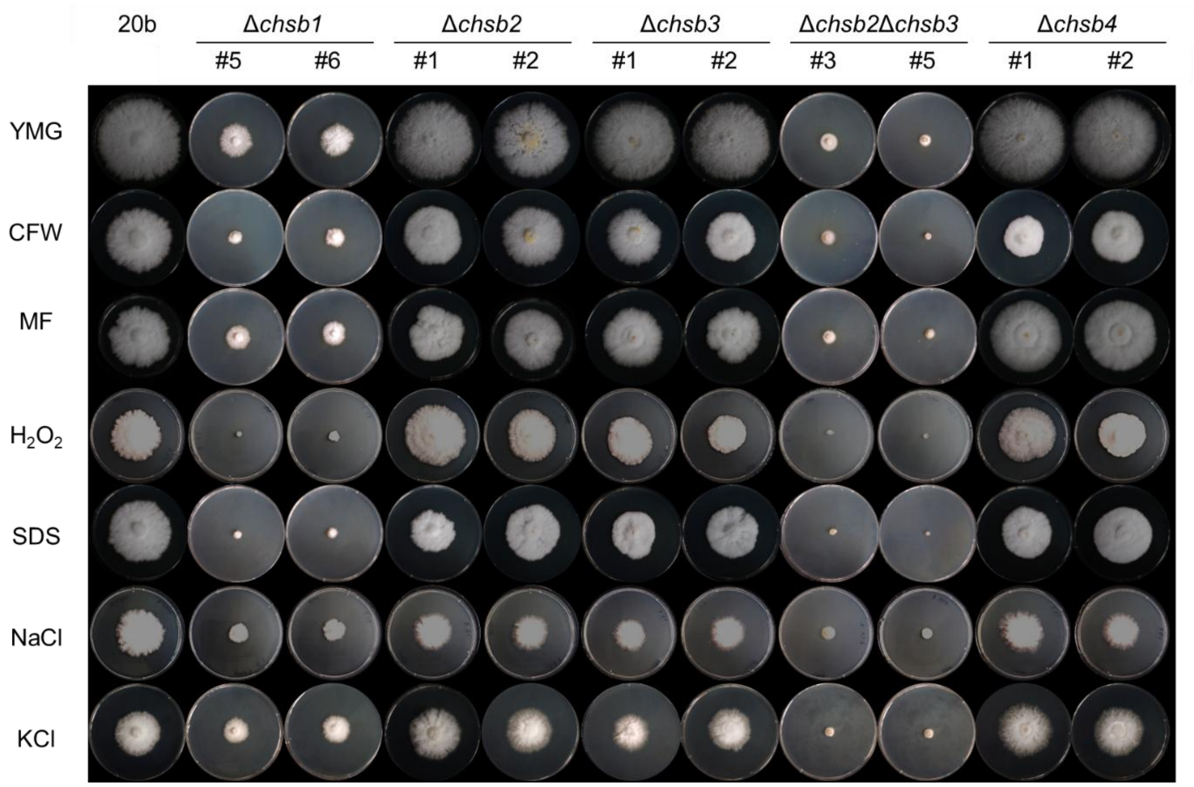

Fig. S5. Phenotypes of basidiomycete specific chitin synthase (*chsb*) gene disruption strains grown on yeast and malt extract with glucose (YMG) agar medium supplemented with 500 μg/mL Calcofluor White (CFW), 100 μg/mL Micafungin (MF), 5 mM H_2O_2, 0.02% sodium dodecyl sulphate (SDS), 0.3 M NaCl, and 0.3 M KCl for 10 d. 20b: parent strain 20b.

Table S1. *P. ostreatus* strains used in this study.

Strain	Genotype/description	Source
PC9	*A2B1*	(3)
20b	*A2B1 ku80*::*cbx*^{R} [a]	(4)
Δ*chsb1*#5	*A2B1 ku80*::*cbx*^{R} *chsb1*::*hph* [b]<br>a *chsb1* disruptant derived from 20b	This study
Δ*chsb1*#6	*A2B1 ku80*::*cbx*^{R} *chsb1*::*hph*<br>a *chsb1* disruptant derived from 20b	This study
Δ*chsb2*#1	*A2B1 ku80*::*cbx*^{R} *chsb2*::*hph*<br>a *chsb1* disruptant derived from 20b	
Δ*chsb2*#2	*A2B1 ku80*::*cbx*^{R} *chsb2*::*hph*<br>a *chsb1* disruptant derived from 20b	(5)
Δ*chsb3*#1	*A2B1 ku80*::*cbx*^{R} *chsb3*::*hph*<br>a *chsb3* disruptant derived from 20b	(5)
Δ*chsb3*#2	*A2B1 ku80*::*cbx*^{R} *chsb3*::*hph*<br>a *chsb3* disruptant derived from 20b	(5)
Δ*chsb2*Δ*chsb3*#3	*A2B1 ku80*::*cbx*^{R} *chsb3*::*hph chsb2*::*bar* [c]<br>a *chsb2chsb3* double disruptant derived from Δ*chsb3*#2	This study
Δ*chsb2*Δ*chsb3*#5	*A2B1 ku80*::*cbx*^{R} *chsb3*::*hph chsb2*::*bar*<br>a *chsb2chsb3* double disruptant derived from Δ*chsb3*#2	This study
Δ*chsb4*#1	*A2B1 ku80*::*cbx*^{R} *chsb4*::*hph*<br>a *chsb3* disruptant derived from 20b	(5)
Δ*chsb4*#2	*A2B1 ku80*::*cbx*^{R} *chsb4*::*hph*<br>a *chsb3* disruptant derived from 20b	(5)

[a] *cbx*^{R} indicates a carboxin resistance gene (6).

[b] *hph* indicates a hygromycin B resistance gene (1).

[c] *bar* indicates a bialaphos resistance gene (2).

Table S2. Primers used for gene disruption of *chsb1* and *chsb2chsb3*

Name	Sequence (5′–3′)
**KS01**	TTCATTTAAACGGCTTCACGGGCAGC
**KS02**	CGCCCTTCAATATTCATCTCTCCATCG
**KS09**	AGTCTTCCGTCGGCTTCTGTCTCCC
**KS10**	GAGTTTGATATGGAGACCCCCAGCC
**KS116**	GCCATTCGTTACTTCATCATCATCG
**KS117**	CGCAAAGATCGAGCATGGATCGCTC
**KS126**	ATGAGCCCAGAACGACGCCCGGCCG
**KS127**	CAGATCTCGGTGACGGGCAGGACCG
**KS14**	GGTGAAATCGTTGCAATCCGGCGG
**KS15**	GCTGCGAGAAATGTAGAGGTCGACG
**KS16**	GCTGATGTTTCCGATAGAGTATAGG
**KS19**	CGCTTCGTTGATAACCCACATATCG
**KS20**	CCCGTCAAGAAGAGCATCAAGCGGG
**KS21**	CTGCCCGTGAAGCCGTTTAAATGAATCGTGGGTCTGTCGACGTGCAGTAC
**KS22**	ATGGAGAGATGAATATTGAAGGGCGAAAGCTGCAGCTCTCGAGACAGCAC
**KS23**	GCAACACATCGTCCCTTGTCCGCTC
**KS28**	GGTCTGAATGACGCGTGACACACTG
**KS32**	CCAGTCTCTAGCTTCGACGTTGACC
**KS33**	GGCAAACAGGAAGCAAGCAATGACG
**KS36**	GGATTCTTCCGGAAGATCTTCCTCC
**KS37**	GGTCTTTAGCCGAACCATTGTCACC
**KS54**	GCTAAGTAGCTGCGGCTCACAGAGC
**KS55**	CCGTCATTGTCAAGGACTTCCAGGG
**KS56**	GCTGCCCGTGAAGCCGTTTAAATGAACTCTGCTCGTCAAGGTTGAAAGTGC
**KS57**	TTCATTTAAACGGCTTCACGGGCAGC
**KS58**	TCCGGTCCTGCCCGTCACCGAGATCTGAGCAAGGAAGTTTGCGGCCAGG
**KS59**	CAGATCTCGGTGACGGGCAGGACC
**KS60**	CCAGATGACATGCCCTCGACGGAGG
**KS61**	GGACGTCCAGATTACACCGTATTGG
**KS64**	CCTCTAGGTATGCTTCGTGTTCTCG
**KS65**	GCAACGAGATACAATCCATACGTCG
**TK203**	ACTCACCGCGACGTCTGTCGAGAAG
**TK204**	CTATTCCTTTGCCCTCGGACGAGTGC

Table S3. Primers used for qRT-PCR.

Protein ID [a]	Gene	Efficiency (%)	Primer name	Primer sequence (5′–3′)
117235	*tub1* (β-tubulin)	96.3	BtubF<br>BtubR	GTGCGTAAGGAAGCTGAGGG<br>TGTGGCATTGTACGGCTCAAC
82171	*ags1*	99.9	YN101<br>YN102	CAAGGTGGTGCCAGATGCTC<br>CGTGTAACCGTGACAGTGTCTGGAG
114314	*fks1*	92.8	YN135<br>YN136	CGAACGCGACGATGGATAGTCATC<br>GGGTAGAGCAGTCCACACTG
114534	*fks2*	114.7	YN137<br>YN138	GAACAACTACGACGCCTATCCTCC<br>GTGGTATTTGCCGCTCTACTGTCC
115957	*chsb1*	88.2	YN119<br>YN120	CTTTGATCAAGCCCAACGTCTGC<br>GGACATCTTGTACTCGAAGTTCTGAG
113050	*chsb2*	96.4	YN113<br>YN114	CGAACCTTCAGGACAAATGTGTTGC<br>CGAGCCAACAAAGCGAACAAAAG
51952	*chsb3*	90.5	TI13<br>TI14	CACATCCAATATGTATCTTGCCGAAG<br>GTATCGTAGCACCCAGTTCGAGTTG
115316	*chsb4*	90.6	YN117<br>YN118	CCGAACAGGAGGCAGCTTCAG<br>GTGTCGGCGTCCACCATTAAG
82871	*chs5*	98.1	YN107<br>YN108	CTTGCCATTCCAGCATTCTCG<br>GTCCCAAAGTTCGTTTTCATATTCGG
81024	*chs6*	98.1	YN105<br>YN106	CAGCTACTGGAAATTATCCTTCGCAG<br>GCAATATTGTCGAAGCAAGAGCG
110811	*chs7*	92.2	YN145<br>YN146	CAAGCCTTATACAACTCCTTCTCGC<br>GCAAGAACAAACTGGAGAGCCAGG
114601	*chs8*	118.8	YN141<br>YN142	GCTTGCTAGAGGGATTACAGG<br>GCGTTCCCGAAGGAGTCTAGGAC
83731	*chs9*	94.3	YN147<br>YN148	GCTGCTATGGTTTCCACTTTCGG<br>CCCTTAGTACCCCACGAAACATCG

[a] Protein ID from the genome database of strain PC9 (JGI *Pleurotus ostreatus* PC9 v1.0, https://genome.jgi.doe.gov/PleosPC9_1/PleosPC9_1.home.html)

References

1. Cummings WJ, Celerin M, Crodian J, Brunick LK, Zolan ME. Insertional mutagenesis in *Coprinus cinereus*: use of a dominant selectable marker to generate tagged, sporulation-defective mutants. *Curr Genet* 1999;36:371-382. https://doi.org/10.1007/s002940050512
2. Avalos J, Geever RF, Case ME. Bialaphos resistance as a dominant selectable marker in *Neurospora crassa*. Curr Genet. 1989;16(5-6):369-72. https://10.1007/bf00340716
3. Larraya LM, Pérez G, Peñas MM, Baars JJ, Mikosch TS, Pisabarro AG, et al. Molecular karyotype of the white rot fungus *Pleurotus ostreatus*. *Appl Environ Microbiol* 1999;65:3413-3417. https://doi.org/10.1128/aem.65.8.3413-3417.1999
4. Salame TM, Knop D, Tal D, Levinson D, Yarden O, Hadar Y. Predominance of a versatile-peroxidase-encoding gene, *mnp4*, as demonstrated by gene replacement via a gene targeting system for *Pleurotus ostreatus*. *Appl Environ Microbiol* 2012;78:5341-5352. https://doi.org/10.1128/aem.01234-12
5. Schiphof K, Kawauchi M, Tsuji K, Yoshimi A, Tanaka C, Nakazawa T, et al. Functional analysis of basidiomycete specific chitin synthase genes in the agaricomycete fungus *Pleurotus ostreatus*. *Fungal Genet Biol* 2024;172:103893. https://10.1016/j.fgb.2024.103893
6. Honda Y, Matsuyama T, Irie T, Watanabe T, Kuwahara M. Carboxin resistance transformation of the homobasidiomycete fungus *Pleurotus ostreatus*. *Curr Genet* 2000;37:209-212. https://doi.org/10.1007/s002940050521
